# Antibiotic treatment for acute sinusitis and subsequent health care use and work absence: a nationwide registry study from Norway

**DOI:** 10.1093/fampra/cmag001

**Published:** 2026-02-07

**Authors:** Marius Skow, Anja Maria Brænd, Louise Emilsson, Sigurd Høye, Jørund Straand, Guro Haugen Fossum

**Affiliations:** The Antibiotic Centre for Primary Care, Department of General Practice, Institute of Health and Society, University of Oslo, Postboks 1130 Blindern, 0318 Oslo, Norway; General Practice Research Unit (AFE), Department of General Practice, Institute of Health and Society, University of Oslo, Postboks 1130 Blindern, 0318 Oslo, Norway; General Practice Research Unit (AFE), Department of General Practice, Institute of Health and Society, University of Oslo, Postboks 1130 Blindern, 0318 Oslo, Norway; Department of Medical Epidemiology and Biostatistics, Karolinska Institutet, PO Box 281, SE-171 77 Stockholm, Sweden; The Antibiotic Centre for Primary Care, Department of General Practice, Institute of Health and Society, University of Oslo, Postboks 1130 Blindern, 0318 Oslo, Norway; General Practice Research Unit (AFE), Department of General Practice, Institute of Health and Society, University of Oslo, Postboks 1130 Blindern, 0318 Oslo, Norway; General Practice Research Unit (AFE), Department of General Practice, Institute of Health and Society, University of Oslo, Postboks 1130 Blindern, 0318 Oslo, Norway

**Keywords:** general practice, anti-bacterial agents, sinusitis, sick leave, cohort studies, health services research

## Abstract

**Background:**

Acute sinusitis is common in general practice. Although typically self-limiting, antibiotics are frequently prescribed despite guideline recommendations to restrict use. It remains unclear whether antibiotic treatment reduces subsequent health care use or work absence.

**Objective:**

To assess how initial treatment with or without antibiotics for acute sinusitis is associated with subsequent health care use and work absence, and to compare phenoxymethylpenicillin (PcV) versus other antibiotics.

**Methods:**

Nationwide registry-based observational cohort study of adults with acute sinusitis (ICPC-2: R75) diagnosed in Norwegian general practice 2012–2019. We compared GP visits, Ear, Nose, and Throat (ENT) specialist visits, repeat antibiotic prescriptions, and sick leave days in antibiotic-treated and untreated episodes. We estimated adjusted differences in outcomes between groups using linear regression (daily outcomes) and negative binomial regression (weekly counts).

**Results:**

We included 627 211 episodes from 413 449 patients. Antibiotics were prescribed in 59% of episodes; 53% received PcV. During the index week, antibiotic use was associated with 1.7 fewer GP visits, 0.1 fewer ENT visits, and 25.1 more sick leave days per 100 episodes. Corresponding figures for the following 4 weeks were: 0.9 fewer GP contacts, 10.7 fewer sick leave days, and 0.6 more antibiotic prescriptions. PcV was associated with slightly more GP visits and re-prescribing than other antibiotics.

**Conclusions:**

Acute sinusitis is followed by a short-term increase in health care use and work absence. Initial antibiotic use was associated with modest short-term differences, but no meaningful reduction in overall follow-up. Findings are consistent with recommendations for restrictive prescribing and narrow-spectrum use when appropriate.

Key messages

- Acute sinusitis was followed by increased health care use.
- Antibiotic-treated episodes had slightly fewer GP and ENT visits early on.
- Antibiotic-treated episodes had more sick leave days during the first week.
- Longer-term outcome differences between treated and untreated episodes were small.
- PcV episodes had slightly more GP visits and re-prescribing than other antibiotics.
- Findings support restrictive and narrow-spectrum prescribing practices.

## Introduction

Acute sinusitis is common and, in most cases, a self-limiting infection without complications [1]. Despite diagnostic uncertainty in distinguishing viral from bacterial sinusitis, there is growing consensus that the potential harms of antibiotic use often outweigh its modest benefits [1, 2]. Internationally, clinical guidelines emphasize restrictive antibiotic use for uncomplicated sinusitis [3, 4]. For example, the American Academy of Otolaryngology recommends watchful waiting as the initial management for most patients, regardless of severity [4].

Nevertheless, antibiotics continue to be widely prescribed for acute sinusitis [5-8]. In Norway, national guidelines recommend limiting use to patients with symptoms persisting for more than 1 week and to use the narrow-spectrum antibiotic phenoxymethylpenicillin (PcV) as first-line treatment [9]. Despite restrictive guidelines, around half of all sinusitis episodes in general practice are treated with antibiotics [10].

Serious complications, such as orbital or intracranial infections requiring hospitalization, are exceptionally rare and not significantly prevented by antibiotics [11]. However, some patients may experience prolonged illness, leading to additional general practitioner (GP) consultations, Ear, Nose, Throat (ENT) specialist referrals, sick leave, or repeat antibiotic prescriptions. How antibiotic treatment affects this extended health care use and work absence has not yet been fully investigated. A better understanding of real-world treatment patterns and their consequences may help inform clinical decision-making and antibiotic stewardship efforts.

In this registry-based study of adult patients with acute sinusitis in Norwegian general practice, we aimed to describe how extended health care use and work absence differ between episodes with and without initial antibiotic treatment. We also explored potential differences between using PcV and other antibiotics.

## Methods

### Study design and setting

We conducted a registry-based observational study comparing extended health care use and work absence for patients with acute sinusitis initially treated with or without antibiotics. We examined two time windows: short-term differences during the index week (Days 0–6 from diagnosis), and longer-term differences over the subsequent 4 weeks (weeks 2–5). The study period was from 1 July 2012 to 30 June 2019.

### Data sources and population

Data from national registries were linked using encrypted personal identifiers. We obtained information on GP and out-of-hours (OOH) contacts (diagnoses, dates) from the Control and Payment of Health Reimbursement Database; specialist and hospital outpatient visits from the Norwegian Patient Register; dispensed antibiotic prescriptions from the Norwegian Prescription Database; and sickness certifications from the Norwegian Labour and Welfare Administration. Demographic and socioeconomic variables were retrieved from Statistics Norway.

Adults aged ≥18 years were eligible if they had a GP or OOH consultation coded as R75 (sinusitis) according to the International Classification of Primary Care, 2nd edition (ICPC-2) [12]. We defined an episode of acute sinusitis as a new R75 diagnosis with no recorded sinusitis consultation in the preceding 90 days and no antibiotic prescription in the preceding 30 days. Each patient could contribute with multiple episodes during the observation period.

### Exposure and outcomes

Antibiotic treatment was defined as a dispensed oral antibiotic within 7 days of the index consultation. In the short-term analyses, only prescriptions on Day 0 or 1 were counted. Antibiotics exclusively used for urinary tract infections (pivmecillinam, trimethoprim, and nitrofurantoin) were not included. Antibiotic-treated episodes were further categorized into those receiving phenoxymethylpenicillin (PcV) or other antibiotics.

The primary outcomes were GP visits, ENT specialist visits, dispensed antibiotics, and days of work absence with physician-issued sickness certification. For the index week, outcomes were measured as mean daily probabilities. In the longer-term analysis (weeks 2–5), outcomes were measured as weekly counts. Initial consultations and index prescriptions were not counted as outcomes. To avoid misclassification due to hospital admissions, we applied a conservative ‘or worse’ algorithm, classifying all outcomes as present if the patient was hospitalized for >24 hours. Follow-up was censored at the time of death.

### Covariates

Estimates were adjusted for sex, age groups, sinusitis risk factors (categorized as: no risk factors, chronic/recurrent sinusitis, and other comorbidities/risk factors), educational level, municipality centrality, and number of episodes per patient. Risk factors were obtained from ICPC-2 codes from GP or OOH contacts during 2012–2019 as well as ICD-10 codes from hospital or specialist contacts in the same period, as described in a previous study [11]. Educational level was categorized as low (primary/lower secondary school), medium (upper secondary school), or high (higher education/university) [13, 14]. Missing educational data were classified as low educational level. Municipal centrality was dichotomized into central (Category 3 in the SSB Centrality index from 2008) [15] and rural (Categories 0, 1, and 2). Work absence with sickness certificates was calculated for patients aged 30–60 to avoid students and retirees.

### Statistical methods

We compared changes in outcomes from a prediagnosis reference period (weeks −8 to −5 before diagnosis) to postdiagnosis periods between the antibiotic and nonantibiotic groups using regression models with an interaction term between treatment status and time period. The interaction term should be interpreted as a contrast in outcome trajectories, facilitating a descriptive comparison of how outcomes change in the two groups over time.

For the short-term analysis, we used linear regression models to estimate mean daily probabilities, with an interaction term between antibiotic treatment and time-period. We opted for linear regression to facilitate direct interpretation of absolute risk differences, given that the outcomes were measured as daily means. Adjusted differences between treatment groups were computed for the cumulative outcome probability for the entire week, with a graphical visualization of adjusted daily differences. For the longer-term analysis, negative binomial regression models were applied to weekly outcome counts due to overdispersion of the data [16]. Adjusted differences over the four-week follow-up period were reported, with graphical presentations of weekly adjusted differences.

Additional analyses compared PcV with other antibiotics using a three-level treatment variable (no antibiotics, PcV, other antibiotics), with interaction terms between the treatment variable and the time-variable.

Estimates are reported as adjusted differences per 100 episodes with 95% confidence intervals (CI). All models were adjusted for calendar month and year to account for seasonality and time trends. Standard errors were clustered at the patient level. To check robustness, we conducted stratified analyses by sex and age group, and sensitivity analyses including only each patient's first sinusitis episode. Analyses were conducted using STATA version 17 (StataCorp).

## Results

### Descriptive data

During the 7-year study period, 413 449 individual patients contributed to a total of 627 211 acute sinusitis episodes in adult patients from an initial 1 192 753 contacts with sinusitis diagnoses. Exclusions were made due to contacts outside the study period (i.e. before July 1st, 2012, or after June 30th 2019: 152 478); prior sinusitis diagnosis in the last 90 days: 373 122; and antibiotic use in the last 30 days: 39 942. Antibiotics were prescribed within seven days in 59% of the episodes. Among these, 93% (343 624) were dispensed within 1 day. Most episodes (84%) occurred in patients without recorded risk factors. Chronic sinusitis was the most common comorbidity, affecting 5% of the patients in the antibiotic group and 9% in the nonantibiotic group. Table 1 shows characteristics at the episode level.

**Table 1:** Descriptive characteristics of episodes of acute sinusitis in Norwegian general practice (2012–2019), comparing episodes with and without antibiotic treatment.

Total	Non-antibiotics		Antibiotics		Total	
	258 153		369 058		627 211	
**Sex**						
Male	84 378	33%	113 112	31%**	197 490	31%
Female	173 775	67%	255 946	69%**	429 721	69%
**Age mean (SD)**	42.7	(15.2)	43.9	(15.4)**	43.4	(15.4)
**Age group**						
18–44	159 744	62%	201 312	55%**	352 056	56%
45–69	93 320	36%	146 377	40%**	239 697	38%
70+	14 089	5%	21 369	6%**	35 458	6%
**Educational level**						
Low	58 603	23%	86 493	23%**	145 096	23%
Medium	103 379	40%	155 643	42%**	259 022	41%
High	96 171	37%	126 922	34%**	223 093	36%
**Centrality**						
Rural	80 646	31%	116 459	32%*	197 105	31%
Urban	177 507	69%	252 599	68%*	430 106	69%
**Risk factors**						
None	212 842	83%	311 683	85%**	524 525	84%
Chronic sinusitis^a^	22 190	9%	19 263	5%**	41 453	7%
Other risk factors^b^	22 714	9%	37 578	10%**	60 292	10%
**Number of episodes**						
Mean (SD)	2.2	(1.6)	2.2	(1.7)**	2.2	(1.6)

Differences between the *Non-antibiotic* and *Antibiotic* groups: *P value <.01. **P values <.001. ^ab^Previous diagnosis of chronic sinusitis, nasal polyps, paranasal sinus surgery, or ≥3 episodes of acute sinusitis in the previous 12 month. ^b^Asthma/COPD, diabetes mellitus, immunocompromised, previous cancer diagnosis, previous opioid abuse diagnosis, head injury/skull malformation [11].

During the 7-year study period, 289 019 (70%) experienced only one episode, while 10 125 patients experienced five or more episodes. These high-frequency patients accounted for almost 10% of the episodes (*n* = 59 624), Supplementary Table 1.

The number of yearly episodes declined over the study period, from 105 196 in the first year to 78 259 in the final year. Over the same period, antibiotic prescribing within 7 days of diagnosis decreased from 68% to 50%. For GP visits, sickness certification, and ENT visits, rates increased incrementally each year. Antibiotic prescribing, on the other hand, showed a consistent decline (Table 2). A clear seasonal variation was observed, with the highest incidence in winter months (December–March) exceeding 60 000 episodes per month, compared with 32 579 and 36 007 in July and August, respectively.

**Table 2:** Acute sinusitis in Norwegian general practice, 2012–2019.

	Study year							
	2012–13	2013–14	2014–15	2015–16	2016–17	2017–18	2018–19	2012–19
**Episodes**	105 196	91 125	96 420	87 855	88 217	80 139	78 259	627 211
Initial prescribing^a^	67.6%	64.2%	62.5%	58.1%	54.5%	50.9%	50.1%	58.8%
PcV proportion^b^	48.7%	49.6%	50.8%	52.3%	56.4%	58.4%	59.1%	52.9%
**Outcome rates**								
Weekly GP visits	20.4	21.5	21.8	22.5	22.7	22.7	23.0	22.0
Weekly ENT visits	0.6	0.6	0.6	0.7	0.7	0.7	0.7	0.6
Weekly days of absence	51.9	53.1	53.6	53.9	53.8	53.6	54.0	53.3
Weekly repeat antibiotic prescriptions	2.6	2.6	2.5	2.4	2.3	2.2	2.2	2.4

Yearly episodes of acute sinusitis and outcome rates for General Practice (GP) contacts, Ear, Nose, Throat (ENT) specialist contacts, sickness certification, and antibiotic prescriptions in. All outcome rates are means per 100 episodes and are calculated from the four-week follow-up period not including the index week.

^a^Antibiotics prescribed within 7 days.

^b^Phenoxymethylpenicillin (PcV) as proportion of all antibiotics.

### Short-term differences

**Figure 1:**
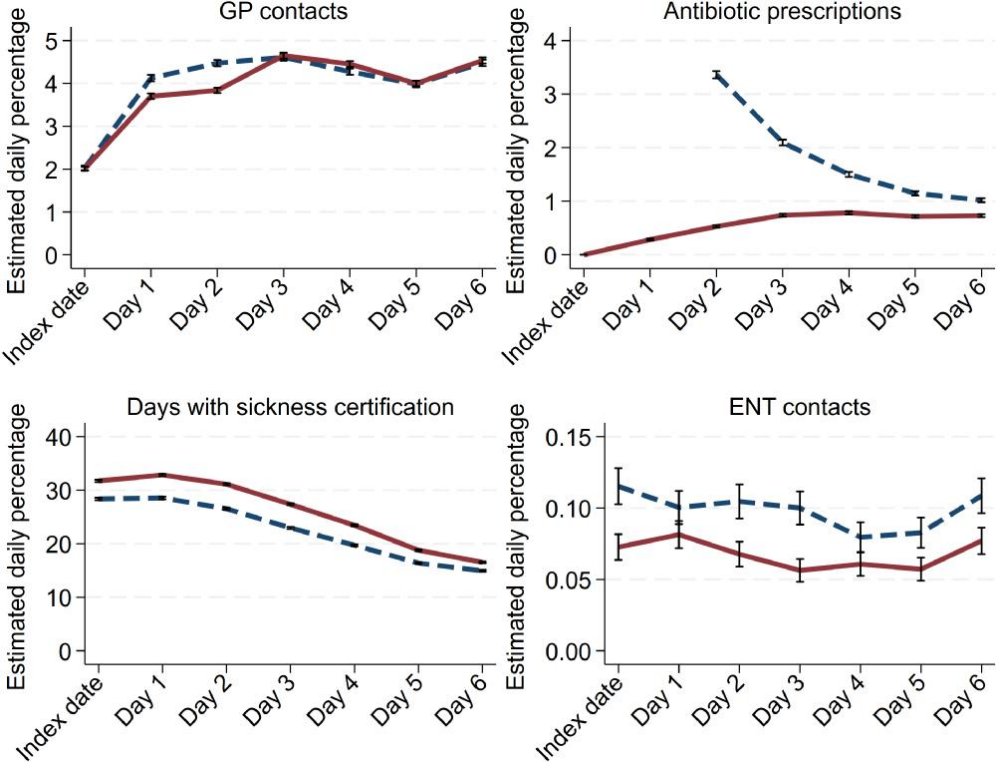
Acute sinusitis in Norwegian general practice 2012–2019 and short-term health care use and work absence. Daily adjusted rates from linear regression comparing episodes initially treated with antibiotics (red line) within 1 day after the index date and those not (blue dashed line). Rates for General Practice (GP) contacts, days with sickness certification, repeat antibiotic prescriptions, and Ear, Nose, Throat (ENT) specialist contacts are presented per 100 episodes. Lines represent estimated daily means; whiskers represent 95% confidence intervals. All estimates are adjusted for age group, sex, educational level, risk factors, centrality, number of episodes per patient, calendar month, and year.

During the index week, all outcome measures increased overall (Fig. 1). Compared with those not treated with antibiotics, patients prescribed antibiotics initially had fewer GP contacts during the index week, with an adjusted difference of −1.7 (95% CI −2.1 to −1.4) visits per 100 episodes. The differences were only evident in the first 3 days.

The likelihood of receiving a further antibiotic prescription was also lower in the antibiotic group, with an adjusted difference of −5.3 (−5.4 to −5.1) prescriptions per 100 episodes during the index week. Sickness certification was more frequent in the antibiotic group with a cumulative adjusted difference of 25.1 (23.5–26.8) more certified sick leave days per 100 episodes during the index week. ENT visits were slightly lower among patients prescribed antibiotics initially with −0.1 (−0.1 to −0.1) fewer visits per 100 episodes during the index week.

When comparing PcV to other antibiotics, the PcV group had 3.9 (3.5–4.4) more GP visits per 100 episodes during the index week and dispensed 0.9 more antibiotic prescriptions per 100 episodes (95% CI 0.8–1.1). Detailed daily rates are available as Supplementary Table 2.

### Longer-term differences

Over the follow-up period (weeks 2–5), all outcome measures initially increased compared to the reference period and then gradually declined (Table 3). The cumulative adjusted difference in GP visits between the antibiotic group and the nonantibiotic group was only −0.9 (−1.7 to −0.1) fewer visits per 100 episodes. Sickness certification showed a difference, with −10.7 (−15.0 to −6.4) days per 100 episodes, indicating fewer certified sick leave days in the antibiotic group.

**Table 3:** Acute sinusitis in Norwegian general practice 2012–2019 and weekly outcome rates, comparing episodes with and without antibiotic treatment.

Weeks	Reference period				Index week	Follow-up period			
	−8	−7	−6	−5	1	2	3	4	5
**GP contacts**									
No antibiotics	15.5	15.6	15.5	15.5	23.7	23.9	21.0	20.0	20.1
Antibiotics	17.1	17.0	17.0	16.7	30.8	26.3	22.5	21.1	20.4
**Days with sickness certification**									
No antibiotics	47.7	47.4	47.4	46.8	154.7	74.3	62.7	60.8	60.4
Antibiotics	48.5	47.7	47.3	46.9	192.0	81.1	58.6	55.2	54.5
**Repeat antibiotic prescriptions**									
No antibiotics	0.9	0.8	0.9	0.8	0.0	3.9	2.2	1.7	1.5
Antibiotics	0.8	0.8	0.8	0.7	3.6	3.7	2.4	2.0	1.7
**ENT contacts**									
No antibiotics	0.4	0.4	0.4	0.4	0.6	0.7	0.8	0.8	0.8
Antibiotics	0.3	0.3	0.3	0.3	0.5	0.5	0.6	0.5	0.6

Adjusted weekly rates from negative binomial regression of the following outcomes: General Practice (GP) contacts, sickness certification days, further antibiotic prescriptions or Ear, Nose, Throat (ENT) specialist contacts.

All values are per 100 episodes and are adjusted for sex, age group, risk factor category, educational level, centrality, number of episodes per patient, calendar month, and year.

**Figure 2:**
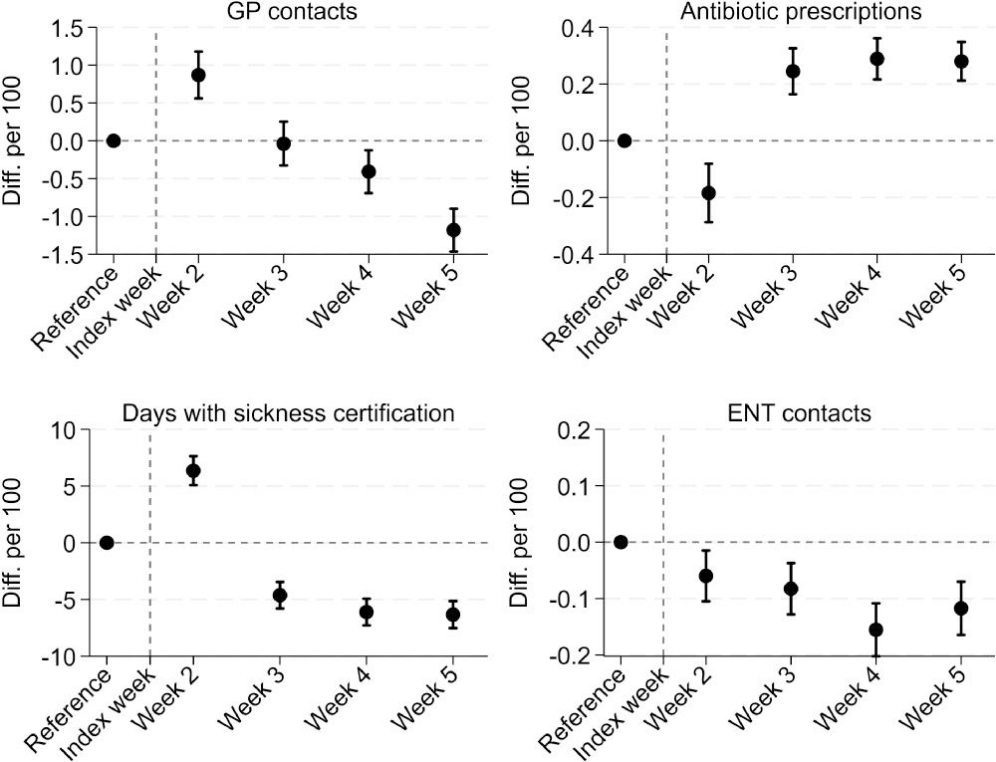
Acute sinusitis in Norwegian general practice 2012–2019 and longer-term health care use and work absence. Estimated weekly differences from negative binomial regressions of outcomes per 100 episodes (diff per 100) comparing episodes treated with antibiotics to those treated without. The figures show adjusted differences for General Practice (GP) contacts, sickness certification days, repeat antibiotic prescriptions, and Ear, Nose, Throat (ENT) specialist contacts across weeks 2–5 after diagnosis. The reference period is weeks −8 to −5 before the index date. Whiskers represent 95% confidence intervals. All estimates are adjusted for age group, sex, educational level, risk factors, centrality, number of episodes per patient, calendar month, and year.

Further antibiotic prescriptions were more common in the antibiotic group, with an adjusted difference of 0.6 (0.4–0.8) more prescriptions per 100 episodes. ENT visits remained infrequent in both groups, with a small difference favouring the antibiotic group (−0.5 visits per 100 episodes; 95% CI −0.6 to −0.4). Weekly differences are presented in Fig. 2.

Among patients receiving antibiotics, those prescribed PcV had higher GP visit rates over the follow-up period compared with those receiving other antibiotics (+3.8 visits per 100 episodes, 95% CI 2.7–4.8; Supplementary Figure 1) and more antibiotic prescriptions (+2.4 per 100 episodes, 95% CI 2.1–2.6). ENT visits and sickness certification did not differ between the PcV and other antibiotic group (−0.1 ENT visits per 100 episodes, 95% CI −0.2 to 0.1; −2.0 certified sick leave days per 100 episodes; 95% CI −7.4 to 3.5). Weekly rates for PcV vs other antibiotics are summarized in Supplementary Table 3.

### Sensitivity analyses

When restricting analyses to only the first-time episodes per patient, findings changed modestly. Antibiotic treatment was associated with more GP visits during the follow-up period (+2.2 per 100 episodes, 95% CI 1.2–3.2), compared to fewer GP visits in the main analysis. The difference in sickness certification was no longer significant (−3.1 per 100 episodes, 95% CI −8.5 to 2.4). Differences in ENT visits (−0.7, 95% CI −0.8 to −0.6), and further prescriptions (+0.2, 95% CI 0.0–0.5) were smaller but remained directionally consistent with the full analysis.

In stratified analyses by age and sex, the direction and magnitude of differences were generally consistent with the main findings. The largest differences in GP contacts and antibiotic prescribing were observed among women aged 67 years or older (Supplementary Figure 2). No consistent difference was observed in ENT contacts across subgroups.

## Discussion

### Key results

In this large retrospective observational study, acute sinusitis was followed by a temporary increase in GP visits, sick leave days, ENT specialist visits, and antibiotic prescriptions. During the first week, patients initially treated with antibiotics had slightly fewer GP contacts, fewer ENT visits, as well as a lower likelihood of receiving additional antibiotic prescriptions, compared to those not prescribed antibiotics. Notably, although antibiotic-treated patients had 25.1 more days per 100 episodes of certified sick leave during the index week, they had 10.7 fewer days per 100 episodes over the subsequent 4 weeks, resulting in only a small net difference over the total period. Other longer-term differences between the antibiotic and nonantibiotic group were also small: the antibiotic group had slightly fewer GP contacts and a marginally higher rate of further antibiotic prescriptions. Among antibiotic-treated patients, those given PcV had marginally higher GP contacts and re-prescribing compared with patients treated with other antibiotics.

### Interpretation

These findings show how patterns of health care use and work absence developed differently in episodes with and without initial antibiotic treatment. The observed short-term reductions in GP and ENT visits in the antibiotic group were small. These differences likely reflect underlying variation in symptom severity, illness duration at presentation, help-seeking behaviour, or clinical decision-making that were not captured in the registry data. Patients prescribed antibiotics may have been more unwell at baseline, explaining their higher number of sick leave days during the index week. Such differences between groups are consistent with confounding by indication [17], where patients who receive antibiotics differ systematically from those not treated in ways that also influence outcomes.

The differences observed should be interpreted as descriptive comparisons of outcome trajectories between treatment groups. Treatment allocation and timing are inherently tied to clinical assessment and evolving symptoms, therefore, the underlying assumptions for causal interpretation of the results—such as parallel trends and strict exogeneity—are unlikely to be met [17].

Clinical practice patterns may also contribute to observed differences. Offering reconsultations rather than immediate antibiotic prescribing aligns with good clinical practice. In many cases, physicians appropriately encourage follow-up visits instead of potentially unnecessary antibiotic prescriptions. Another GP strategy could be to refer to an ENT specialist rather than to treat with antibiotics. The slightly higher ENT visits in the nonantibiotic group might reflect such a strategy, or it might be caused by a higher share of patients with chronic sinusitis in the nonantibiotic group. GP visits, antibiotic prescribing, and work absence are not solely determined by patient symptoms, as these outcomes often reflect a complex interplay of clinical judgement, patient expectations, and organizational factors [18]. Other possible explanations for our findings are that GP visits inherently may increase the probability of more healthcare use [19, 20]. Work absence itself is likely underestimated, as most employees in Norway have three days of self-certification [21].

Our sensitivity analysis, restricted to first-time episodes per patient, revealed slightly different patterns: antibiotic-treated patients had more GP contacts during follow-up, and differences in sick leave days were no longer statistically significant. This suggests that patients with recurrent episodes may represent a subgroup with more persistent symptoms and/or a lower threshold for seeking care. Presenting both overall and first-episode results offers a more complete picture of how patients are managed in general practice and highlights the influence of patient-level characteristics on extended health care use and work absence.

### Strengths and limitations

This study benefits from comprehensive national registry data capturing all GP, OOH, and specialist consultations, prescriptions, and sickness certification within Norway's publicly funded healthcare system. This allowed for a broad assessment of extended health care use and work absence following acute sinusitis. Our analytic strategy allows for exploring patient trajectories of patients with acute sinusitis on several levels of health care. We also adjusted for calendar month and year to account for seasonal variation and temporal prescribing trends.

A limitation of the dataset is the lack of detailed clinical information. GP visits often involve multiple issues, making it difficult to isolate contacts specifically related to sinusitis. A British study found that an average GP consultation involves 2–3 problems, with only 37% coded appropriately [22], highlighting a potential limitation of using reimbursement codes for inclusion. Diagnosing acute bacterial sinusitis in general practice is challenging, and the ICPC-2 coding system introduces further uncertainty to diagnosis precision [2, 23, 24]. However, research suggests that diagnostic coding in Norwegian general practice is at an acceptable level [25]. Our decision to use R75 (sinusitis) as the primary inclusion criterion ensures a more homogeneous study population, as including broader respiratory codes could introduce additional complexity and uncertainty. We applied a 90-day washout period to avoid capturing chronic sinusitis and added a sensitivity analysis to highlight observations caused by patients with chronic or recurring sinusitis.

### Implications

This study provides real-world insight into acute sinusitis and subsequent use of health services and work absence. The small differences we observed between those treated with antibiotics and those not treated may indicate that reducing antibiotic prescribing for acute sinusitis is unlikely to result in clinically meaningful increases in extended health care use or longer sick leave. These findings may reassure clinicians that restrictive antibiotic prescribing is unlikely to increase their follow-up workload. From a stewardship perspective, this supports existing guideline recommendations promoting restrictive use and reliance on narrow-spectrum antibiotics such as PcV when antibiotics are deemed necessary.

Our results align with evidence from randomized trials and systematic reviews on acute sinusitis. In a Cochrane review, nearly half of patients were symptom-free after 1 week without antibiotics (rising to about two-thirds by 14 days), and antibiotics only reduced symptom duration in a fraction of cases [8]. Similarly, a systematic review [26] noted that antibiotics only improve early symptom relief at Days 3–7 but by Day 10 there was no difference in outcomes between the antibiotic and placebo groups.

Future studies should also aim to include clinical or patient-reported data, particularly symptom severity, recovery trajectories, and adverse events. Combining registry and clinical data could help identify a subgroup of patients most likely to benefit from antibiotics and guide more targeted treatment strategies.

## Conclusion

Acute sinusitis in general practice is followed by a short-term increase in extended health care use and work absence. Patients initially treated with antibiotics had modestly reduced rates of follow-up GP consultations and ENT visits, but also slightly higher further antibiotic use and only marginal differences in sickness certification. Overall, our findings support the safety of restrictive antibiotic use for acute sinusitis and support efforts to further reduce unnecessary antibiotic prescribing.

## Supplementary Material

cmag001_Supplementary_Data

## Data Availability

The data for this article were provided by third parties by permission and are not publicly available due to privacy legislations. Data may be requested from data owners upon ethical clearance.

## References

[1] Fokkens WJ, Lund VJ, Hopkins C et al European position paper on rhinosinusitis and nasal polyps 2020. Rhinology 2020;58:1–464. 10.4193/Rhin20.600 32077450

[2] Autio TJ, Koskenkorva T, Närkiö M et al Diagnostic accuracy of history and physical examination in bacterial acute rhinosinusitis. Laryngoscope 2015;125:1541–6. 10.1002/lary.25247 25782075

[3] National Institute for Health and Care Excellence . Sinusitis (acute): antimicrobial prescribing. 2017. https://www.nice.org.uk/guidance/ng79.

[4] Rosenfeld RM, Piccirillo JF, Chandrasekhar SS et al Clinical practice guideline (update): adult sinusitis. Otolaryngol Head Neck Surg 2015;152:S1–S39. 10.1177/0194599815572097 25832968

[5] Schwartz KL, Langford BJ, Daneman N et al Unnecessary antibiotic prescribing in a Canadian primary care setting: a descriptive analysis using routinely collected electronic medical record data. CMAJ Open 2020;8:E360–e69. 10.9778/cmajo.20190175 PMC7207032 32381687

[6] Smieszek T, Pouwels KB, Dolk FCK et al Potential for reducing inappropriate antibiotic prescribing in English primary care. J Antimicrob Chemother 2018;73:ii36–ii43. 10.1093/jac/dkx500 29490058 PMC5890667

[7] Kasse GE, Cosh SM, Humphries J et al Antimicrobial prescription pattern and appropriateness for respiratory tract infection in outpatients: a systematic review and meta-analysis. Syst Rev 2024;13:229. 10.1186/s13643-024-02649-3 39243046 PMC11378372

[8] Lemiengre MB, van Driel ML, Merenstein D et al Antibiotics for acute rhinosinusitis in adults. Cochrane Database Syst Rev 2018:1–80. 10.1002/14651858.CD006089.pub5 PMC6513448 30198548

[9] Norwegian Directorate of Health . National Guidelines for Antibiotics in Primary Health Care. 2021. https://www.helsedirektoratet.no/retningslinjer/antibiotika-i-primaerhelsetjenesten/infeksjoner-i-ovre-luftveier#akutt-sinusitt.

[10] Skow M, Fossum GH, Høye S et al Antibiotic treatment of respiratory tract infections in adults in Norwegian general practice. JAC Antimicrob Resist 2022;5. 10.1093/jacamr/dlac135 PMC9825809 36632357

[11] Skow M, Fossum GH, Høye S et al Hospitalizations and severe complications following acute sinusitis in general practice: a registry-based cohort study. J Antimicrob Chemother 2023;78:2217–27. 10.1093/jac/dkad227 37486144 PMC10477136

[12] World Health Organization . International Classification of Primary Care, 2nd edition (ICPC-2). 2003. https://www.who.int/standards/classifications/other-classifications/international-classification-of-primary-care.

[13] Amundsen O, Moger TA, Holte JH et al Combination of health care service use and the relation to demographic and socioeconomic factors for patients with musculoskeletal disorders: a descriptive cohort study. BMC Health Serv Res 2023;23:858. 10.1186/s12913-023-09852-3 37580723 PMC10426198

[14] Riiser S, Haukenes I, Baste V et al Variation in general practitioners' depression care following certification of sickness absence: a registry-based cohort study. Fam Pract 2021;38:238–45. 10.1093/fampra/cmaa120 33152060 PMC8211146

[15] Statistics Norway . Classification of centrality—Centrality 2008. 2008. https://www.ssb.no/en/klass/klassifikasjoner/128/versjon/468/koder.

[16] Hilbe JM . Overdispersion. In: Hilbe JM (ed.) Negative Binomial Regression, 2nd ed. Cambridge: Cambridge University Press, 2011, 141–84.

[17] Lash TL, Lash TL, VanderWeele T et al Modern Epidemiology, 4th edi. Philadelphia: Wolters Kluwer, 2021, 911–2.

[18] Kiessling A, Arrelöv B. Sickness certification as a complex professional and collaborative activity—a qualitative study. BMC Public Health 2012;12:702. 10.1186/1471-2458-12-702 22928773 PMC3499228

[19] Weinberger M, Oddone EZ, Henderson WG. Does increased access to primary care reduce hospital readmissions? Veterans Affairs Cooperative Study Group on Primary Care and Hospital Readmission. N Engl J Med 1996;334:1441–7. 10.1056/nejm199605303342206 8618584

[20] Starfield B . Is primary care essential? The Lancet 1994;344:1129–33. doi: 10.1016/S0140-6736(94)90634-3 7934497

[21] Norwegian Ministry of Labour and Social Affairs . The IA Agreement 2014–2018. 2014. https://www.regjeringen.no/globalassets/departementene/aid/dokumenter/2016/ia_agreement_-2014_18.pdf.

[22] Salisbury C, Procter S, Stewart K et al The content of general practice consultations: cross-sectional study based on video recordings. British Journal of General Practice 2013;63:e751–e59. 10.3399/bjgp13X674431 PMC3809428 24267858

[23] Ebell MH, McKay B, Dale A et al Accuracy of signs and symptoms for the diagnosis of acute rhinosinusitis and acute bacterial rhinosinusitis. Ann Fam Med 2019;17:164–72. 10.1370/afm.2354 30858261 PMC6411403

[24] Hornung CM, Ganti A, Lunos S et al Characterizing trends in diagnosis and management of sinusitis in a large health care system: from primary care to otolaryngology. Ann Otol Rhinol Laryngol 2024;133:476–484. 10.1177/00034894241230365 38345045

[25] Sporaland GL, Mouland G, Bratland B et al General practitioners' use of ICPC diagnoses and their correspondence with patient record notes. Tidsskr Nor Laegeforen 2019;139:1468–72. 10.4045/tidsskr.18.0440 31642635

[26] Burgstaller JM, Steurer J, Holzmann D et al Antibiotic efficacy in patients with a moderate probability of acute rhinosinusitis: a systematic review. Eur Arch Oto-Rhino-Laryngol 2016;273:1067–77. 10.1007/s00405-015-3506-z 25597034

