# Supplementary material for: Antibiotic treatment for acute sinusitis and subsequent health care use and work absence: a nationwide registry study from Norway
Source: Fam Pract. 2026 Feb 7;43(2):cmag001. doi: 10.1093/fampra/cmag001 (PMC13017361; doi:10.1093/fampra/cmag001)

Supplementary material

Supplementary table 1. Descriptive characteristics of episodes of acute sinusitis in Norwegian general practice (2012–2019), comparing episodes with and without antibiotic treatment. Only episodes in patients with 5 or more episodes.

	Non-antibiotics		Antibiotics		Total	
**Total**	24 404		35 220		59 624	
**Sex**						
Male	6 588	27%	7 632	22%**	14 220	24%
Female	17 816	73%	27 588	78%**	45 404	76%
**Age** mean (SD)	44.9	13.6	45.2	13.5*	45.1	13.5
**Age group**						
18-44	12 726	52%	17 877	51%**	30 603	51%
45-69	10 544	43%	15 942	45%**	26 486	44%
70+	1 134	5%	1 401	4%**	2 535	4%
**Educational level**						
Low	4 803	20%	7 881	22%**	12 684	21%
Medium	10 285	42%	15 306	43%**	25 591	43%
High	9 316	38%	12 033	34%**	21 349	36%
**Centrality**						
Rural	7 601	31%	11 424	32%**	19 025	32%
Urban	16 803	69%	23 796	68%**	40 599	68%
**Risk factors**						
None	14 782	61%	23 171	66% **	37 953	64%
Chronic sinusitis †	7 025	29%	7 102	20% **	14 127	24%
Other risk factors ‡	2 569	11%	4 872	14% **	7 441	13%
**Number of episodes**						
Mean (SD)	6.1	1.3	6.2	1.5**	6.1	1.4

* P value < 0.01. ** P values <0.001. † b Previous diagnosis of chronic sinusitis, nasal polyps, paranasal sinus surgery, or ≥3 episodes of acute sinusitis in the previous 12 month. ‡ Asthma/COPD, diabetes mellitus, immunocompromised, previous cancer diagnosis, previous opioid abuse diagnosis, head injury/skull malformation.

Supplementary table 2. Acute sinusitis in Norwegian general practice 2012-2019 and short-term health care use and work absence. Daily adjusted rates from linear regressions of General Practice (GP) contacts, sickness certification days, further antibiotic prescriptions, and Ear, Nose, Throat (ENT) specialist contacts per 100 episodes. Rates are presented by antibiotic group (no antibiotics, phenoxymethylpenicillin [PcV], or other antibiotics), defined by dispensing within 1 day after the index date.

	Reference	Index date	Day 1	Day 2	Day 3	Day 4	Day 5	Day 6
**GP visits**								
No antibiotics	2.3	2.0	4.1	4.5	4.6	4.3	4.0	4.5
PcV	2.3	2.3	3.6	4.0	4.9	4.7	4.2	4.6
Other antibiotics	2.5	1.7	3.8	3.7	4.4	4.2	3.8	4.4
**Sickness certification**								
No antibiotics	6.9	28.3	28.5	26.5	22.9	19.7	16.4	14.9
PcV	6.9	32.1	33.2	31.6	27.8	23.9	19.1	16.9
Other antibiotics	6.7	31.3	32.4	30.6	26.9	22.9	18.4	16.1
**Further antibiotic prescriptions**								
No antibiotics	0.1	0.0	0.0	3.4	2.1	1.5	1.1	1.0
PcV	0.1	0.0	0.2	0.5	0.8	0.9	0.8	0.8
Other antibiotics	0.1	0.0	0.4	0.6	0.7	0.7	0.6	0.7
**ENT visits**								
No antibiotics	0.06	0.12	0.10	0.10	0.10	0.08	0.08	0.11
PcV	0.04	0.05	0.06	0.06	0.05	0.05	0.05	0.06
Other antibiotics	0.05	0.10	0.10	0.08	0.07	0.07	0.06	0.09

All values are per 100 episodes, and are adjusted for sex, age group, risk factors, educational level, geographical centrality and patient number of episodes during the study period.

Supplementary table 3. Acute sinusitis in Norwegian general practice 2012-2019 and weekly outcome rates from negative binomial regression, comparing episodes treated by phenoxymethylpenicillin [PcV], with other antibiotics, defined by dispensing within 7 days. Adjusted weekly rates from negative binomial regression of the following outcomes: General Practice (GP) contacts, sickness certification days, further antibiotic prescriptions or Ear, Nose, Throat (ENT) specialist contacts.

	Reference period				Index	Follow-up period			
Weeks	-8	-7	-6	-5	1	2	3	4	5
**GP contacts**									
PcV	16.2	16.2	16.3	16.0	31.6	26.3	22.0	20.6	19.6
Other antibiotics	18.0	18.0	17.8	17.6	29.9	26.3	23.1	21.7	21.2
**Days with sickness certification**									
PcV	48.6	47.9	47.7	47.2	196.2	82.2	58.5	54.9	54.2
Other antibiotics	48.6	47.7	47.0	46.7	184.6	79.5	58.5	55.5	54.8
**Antibiotic prescriptions**									
PcV	0.6	0.6	0.6	0.5	3.8	4.0	2.4	1.9	1.6
Other antibiotics	1.0	1.1	1.1	0.9	3.4	3.5	2.4	2.1	1.9
**ENT contacts**									
PcV	0.2	0.3	0.3	0.3	0.4	0.5	0.5	0.5	0.5
Other antibiotics	0.4	0.4	0.3	0.3	0.7	0.6	0.6	0.6	0.6

All rates are per 100 episodes, and are adjusted for sex, age group, risk factor category, educational level, centrality, number of episodes per patient, calendar month, and year.

Supplementary figure 1. *Acute sinusitis in Norwegian general practice 2012-2019 and longer-term health care use and work absence. Estimated weekly differences from negative binomial regressions of outcomes per 100 episodes (diff per 100) comparing episodes treated with phenoxymethylpenicillin (green filled circles) and other antibiotics (red hollow circles) with no antibiotics (dashed horizontal line). The figures show adjusted differences for General Practice (GP) contacts, sickness certification days, repeat antibiotic prescriptions, and Ear, Nose, Throat (ENT) specialist contacts across weeks 2 to 5 after diagnosis. The reference period is weeks -8 to -5 before index date. Whiskers represent 95% confidence intervals. All estimates are adjusted for age group, sex, educational level, risk factors, centrality, number of episodes per patient, calendar month, and year*

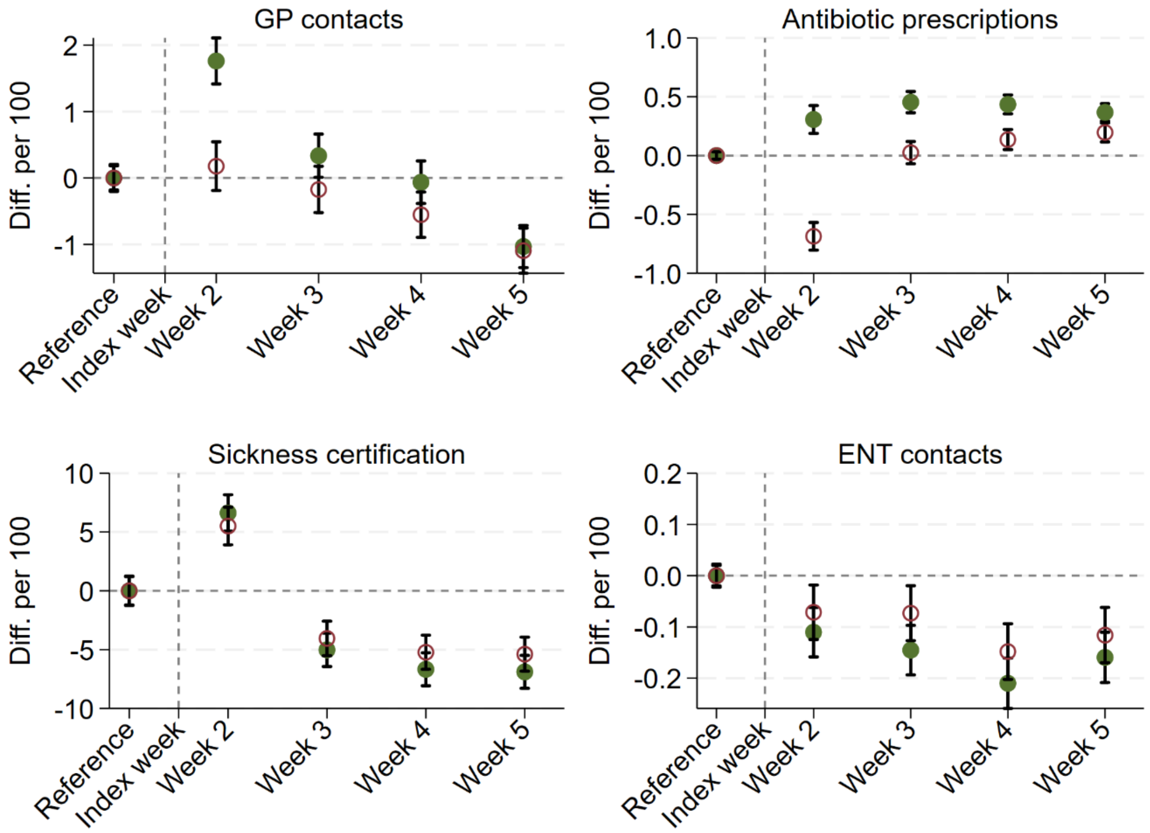

Supplementary figures 2a-b. *Acute sinusitis in Norwegian general practice 2012-2019 and longer-term health care use and work absence stratified by sex and age group. Estimated weekly differences from negative binomial regressions of GP visits (Figure S2a) and repeat antibiotic prescribing (Figure S2b) per 100 episodes comparing episodes treated with antibiotics to those treated without. The reference period is weeks -8 to -5 before index date. Whiskers represent 95% confidence intervals. All estimates are adjusted for age group, sex, educational level, risk factors, centrality, number of episodes per patient, calendar month, and year.*

2a:

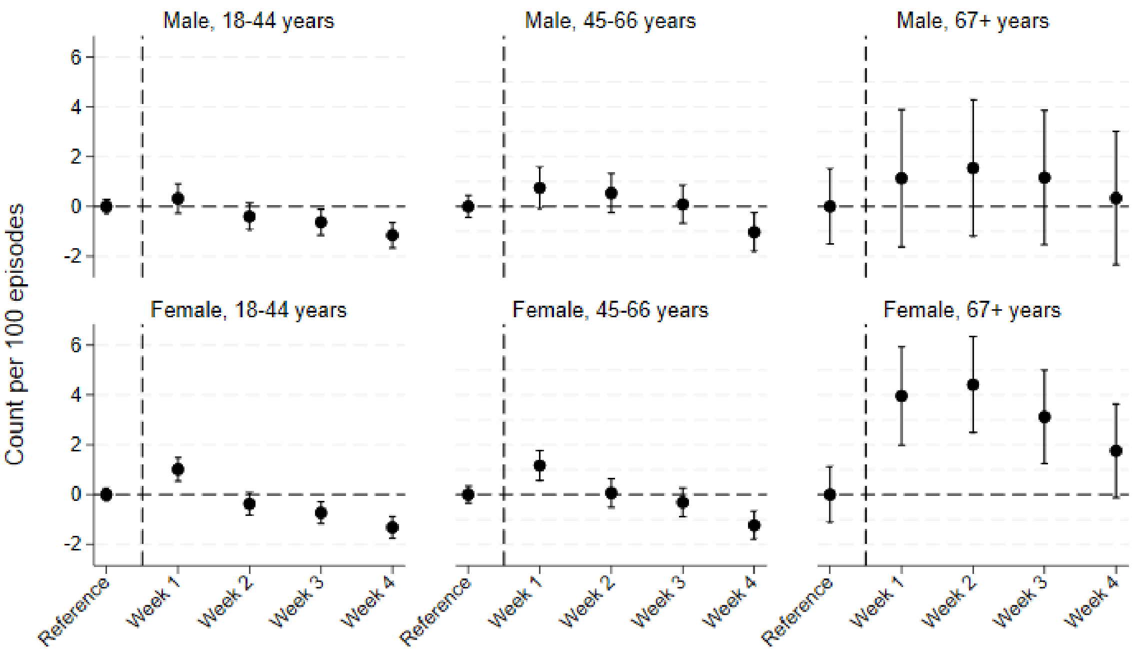

2b:

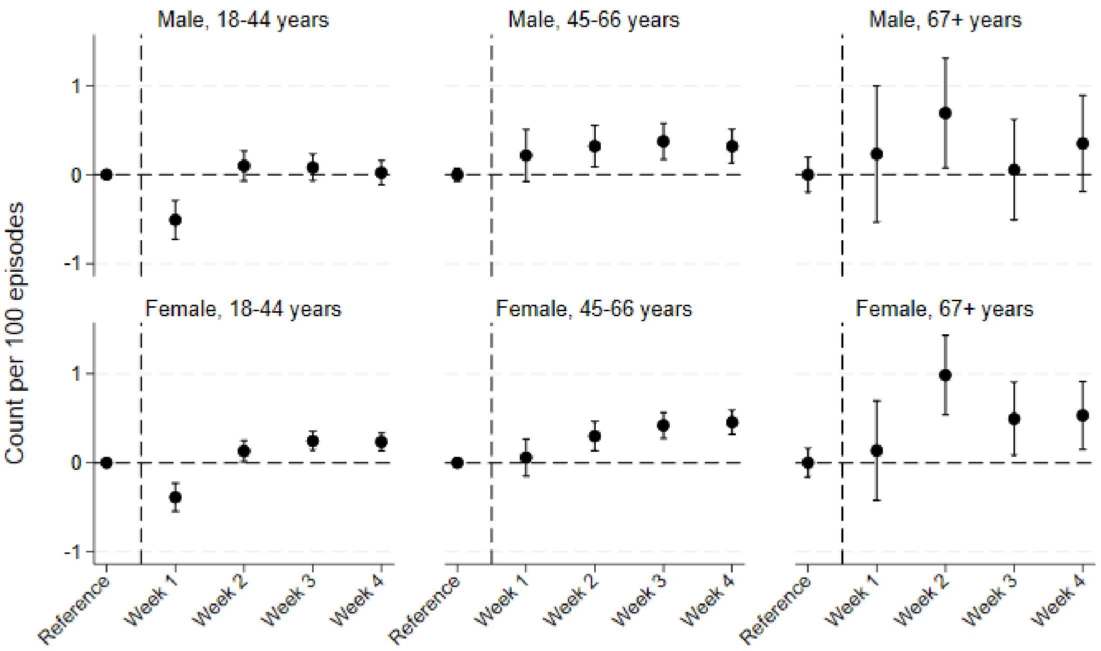

Supplement: cmag001_Supplementary_Data [file cmag001_supplementary_data.pdf]
